# Using the Textual Content of Radiological Reports to Detect Emerging Diseases: A Proof-of-Concept Study of COVID-19

**DOI:** 10.1007/s10278-023-00949-z

**Published:** 2024-01-12

**Authors:** Amandine Crombé, Jean-Christophe Lecomte, Mylène Seux, Nathan Banaste, Guillaume Gorincour

**Affiliations:** 1 IMADIS, Lyon, France; 2 SARCOTARGET Team, University of Bordeaux, Inserm, UMR1312, BRIC, BoRdeaux Institute of Oncology, 146 Rue Léo Saignat, Bordeaux, F-33076 France; 3 Department of Radiology, Pellegrin University Hospital, CHU Bordeaux, Place Amélie Raba-Léon, Bordeaux, F-33076 France; 4 Centre Aquitain d’Imagerie médicale, Mérignac, France; 5 Centre Hospitalier de Saintes, Saintes, France; 6 Clinique Mutualiste Bordeaux Pessac, Pessac, France; 7 Clinique Convert, Ramsay, Bourg en Bresse, France; 8 Clinique Bouchard, ELSAN, Marseille, France

**Keywords:** Natural language processing, Computed tomography, Radiological reports, SARS-CoV-2, Coronavirus disease 2019, Time series analysis, Unsupervised clustering

## Abstract

Changes in the content of radiological reports at population level could detect emerging diseases. Herein, we developed a method to quantify similarities in consecutive temporal groupings of radiological reports using natural language processing, and we investigated whether appearance of dissimilarities between consecutive periods correlated with the beginning of the COVID-19 pandemic in France. CT reports from 67,368 consecutive adults across 62 emergency departments throughout France between October 2019 and March 2020 were collected. Reports were vectorized using time frequency–inverse document frequency (TF-IDF) analysis on one-grams. For each successive 2-week period, we performed unsupervised clustering of the reports based on TF-IDF values and partition-around-medoids. Next, we assessed the similarities between this clustering and a clustering from two weeks before according to the average adjusted Rand index (AARI). Statistical analyses included (1) cross-correlation functions (CCFs) with the number of positive SARS-CoV-2 tests and advanced sanitary index for flu syndromes (ASI-flu, from open-source dataset), and (2) linear regressions of time series at different lags to understand the variations of AARI over time. Overall, 13,235 chest CT reports were analyzed. AARI was correlated with ASI-flu at lag = + 1, + 5, and + 6 weeks (*P* = 0.0454, 0.0121, and 0.0042, respectively) and with SARS-CoV-2 positive tests at lag = − 1 and 0 week (*P* = 0.0057 and 0.0001, respectively). In the best fit, AARI correlated with the ASI-flu with a lag of 2 weeks (*P* = 0.0026), SARS-CoV-2-positive tests in the same week (*P* < 0.0001) and their interaction (*P* < 0.0001) (adjusted *R*^2^ = 0.921). Thus, our method enables the automatic monitoring of changes in radiological reports and could help capturing disease emergence.

**Supplementary Information:**

The online version contains supplementary material available at 10.1007/s10278-023-00949-z.

## Introduction

Radiological reports represent a colossal amount of information with several applications. Using natural language processing (NLP) to label reports could help generate large cohorts, plan human and technical resources, assess compliance with guidelines, and detect discrepancies between results and conclusions [1–3]. It has been recently shown that the structure and content of reports developed by emergency radiologists depend on their personal background, examination characteristics, or workload [4]. On a clinical side, one could hypothesize that an emerging new disease with significant impact on health would lead to new patterns of radiological depictions that could be captured with NLP before the semiology of the disease has been deciphered, which is inherently shifted by several weeks due to the time needed to understand patterns, collect databases, and statistically verify associations between features and diseases. Thus, such NLP-based detection methods on radiological reports could complement other efforts to detect emerging new disease notably wastewater-based surveillance in addition to clinical surveillance [5, 6].

Regarding the coronavirus disease 2019 (COVID-19) outbreak due to severe acute respiratory syndrome coronavirus 2 (SARS-CoV-2), the first patients were clinically reported in China in December 2019 [7]. The first radiological series involving the initial strain was published online in February 2020 and highlighted peculiar semiology on chest CT with bilateral peripheral ground glass opacities (GGOs), consolidations, and interstitial thickening [8, 9]. In France, the first three patients were identified on January 24, 2020, followed by progressive spread in the French territory until the first French lockdown on March, 17, 2020 (with *n* = 1097 patients newly diagnosed with positive SARS-CoV-2 by reverse transcriptase polymerase chain reaction (RT-PCR)) [10]. The French Society of Radiology and the French Society of Thoracic Imaging (SFR-SIT) actively provided templates for standardized chest CT reports in the setting of suspected SARS-CoV-2 infection across the radiologist community on April 1, 2020 [11]. Between the first COVID-19 diagnosis in France and the availability of these templates, French radiologists wrote their reports according to their own experience in thoracic imaging and the objective abnormalities on chest CT. So far, most studies using artificial intelligence have applied a supervised methodology on medical images in order to perform patients’ triage, distinguishing common pneumonitis from COVID-19 lung disease, assessing the severity of the COVID-19 lung disease, or anticipating oxygen requirement thanks to classical machine-learning or deep-learning algorithms [12–16]. Regarding NLP application, Li et al. trained supervised machine-learning models to automatically identify CT reports with the diagnosis of acute appendicitis, diverticulitis, and bowel obstruction and secondarily applied those models on a large population to investigate the impact of the COVID-19 pandemic on their detection in emergency departments [17].

Consequently, our aims were (i) to develop an original unsupervised NLP method to detect variations in the content of chest CT reports at a population (or macroscopic) scale, without a priori knowledge of the possible occurrence of a new disease and its typical radiological presentation and before the availability of biological diagnostic tests for the whole population, and (ii) to test the ability of this method to detect the start of the COVID-19 pandemic in France.

## Materials and Methods

### Study Design and Population

This observational retrospective multicenter study was approved by the French national radiological review board (CRM-2303–337). The need for written informed consent was waived due to its retrospective nature and to the fact that data were anonymized.

Three cohorts from IMADIS Teleradiology were investigated: Cohort-1 (covering the 4 months before the first official COVID-19 case in France to 2 weeks after the 1st French lockdown) and two reference cohorts named Cohort-R1 (covering the first 2 weeks of September 2019, i.e., distant from any potential event) and Cohort-R2 (covering the first 4 weeks of November 2020, i.e., during the peak of the 2nd COVID-19 wave in France). IMADIS Teleradiology is a medical company dedicated to the remote interpretation of imaging from emergency departments in French public and private hospitals.

In Cohort-1, we included all consecutive patients between October 6, 2019, and March 28, 2020, who fulfilled the following criteria: (i) had a request for a CT of at least the chest by an emergency physician from one of the 62 partner centers of IMADIS Teleradiology at that time and (ii) had an available radiological report made in real time by one of the 171 emergency radiologists working at IMADIS Teleradiology during this study period.

In Cohort-R1, the same inclusion criteria were applied to patients between September 1, 2019, and September 14, 2019.

In Cohort-R2, we included all consecutive patients between November 1, 2020, and November 28, 2020, who fulfilled the following criteria: (i) had a request for a CT of only the chest by an emergency physician from one of the 76 partner centers of IMADIS Teleradiology at the time, and (ii) had an available radiological report made in real-time by one of the 173 emergency radiologists working at IMADIS Teleradiology at that time. The rationale for excluding examinations not specifically covering the chest in Cohort-R2 was to obtain a representative cohort of examinations that were more likely to be specifically requested for COVID-19 during a period of high prevalence of positive SARS-CoV-2 tests.

For all cohorts, we excluded patients with denied requests, MRIs, secondary opinions from outside center examinations, radiological reports not containing a clearly defined “Results” section, CTs involving body areas other than the chest, no clearly defined paragraph for the chest analysis within the “Results” section (for instance, starting with a heading such as “Thorax,” “Chest,” or “Thoracic analysis,” and finishing with a line break).

**Fig. 1:**
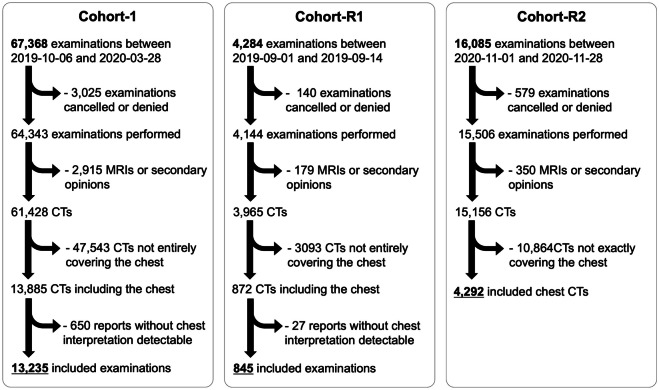
Study flowcharts. Abbreviations: CT, computed tomography; MRI, magnetic resonance imaging

Figure 1 shows the flowchart.

### Text Preprocessing

The radiological reports were written in French. Radiologists completed free-text areas by typing or using speech recognition software (Dragon Medical Direct, Nuance Healthcare, Burlington, MA, USA). Spelling mistakes were highlighted in real time to reduce manual corrections. Templates for normal examinations were available and editable. Regarding the Cohort-R2, structured reports for the analysis of chest CT for suspected COVID-19 were also available based on the template provided by the SFR-SIT on April 2020.

Text preprocessing was performed with R (v.4.1.0, The R foundation for Statistical Computing, Vienna, Austria) using the “tidytext” and “stringr” packages [18] and focused on the paragraph related to chest analysis in the “Results” section, as these results were the most meaningful and likely to be modified based on new radiological findings. Supplemental Data S1 details the preprocessing.

### Iterative Unsupervised Clustering

Our aim was to automatically perform unsupervised clustering of the preprocessed reports over consecutive biweekly periods (T) and to compare the similarity of the resulting clusters from the clustering of a reference period two weeks before (T-2). It must be emphasized that the accuracy of the depictions in the chest CT reports was not specifically verified in this pipeline and that there was no supervised analysis with an outcome to predict. In other words, herein, our goal was to classify the texts without any a priori depending on the words they contain.

**Fig. 2:**
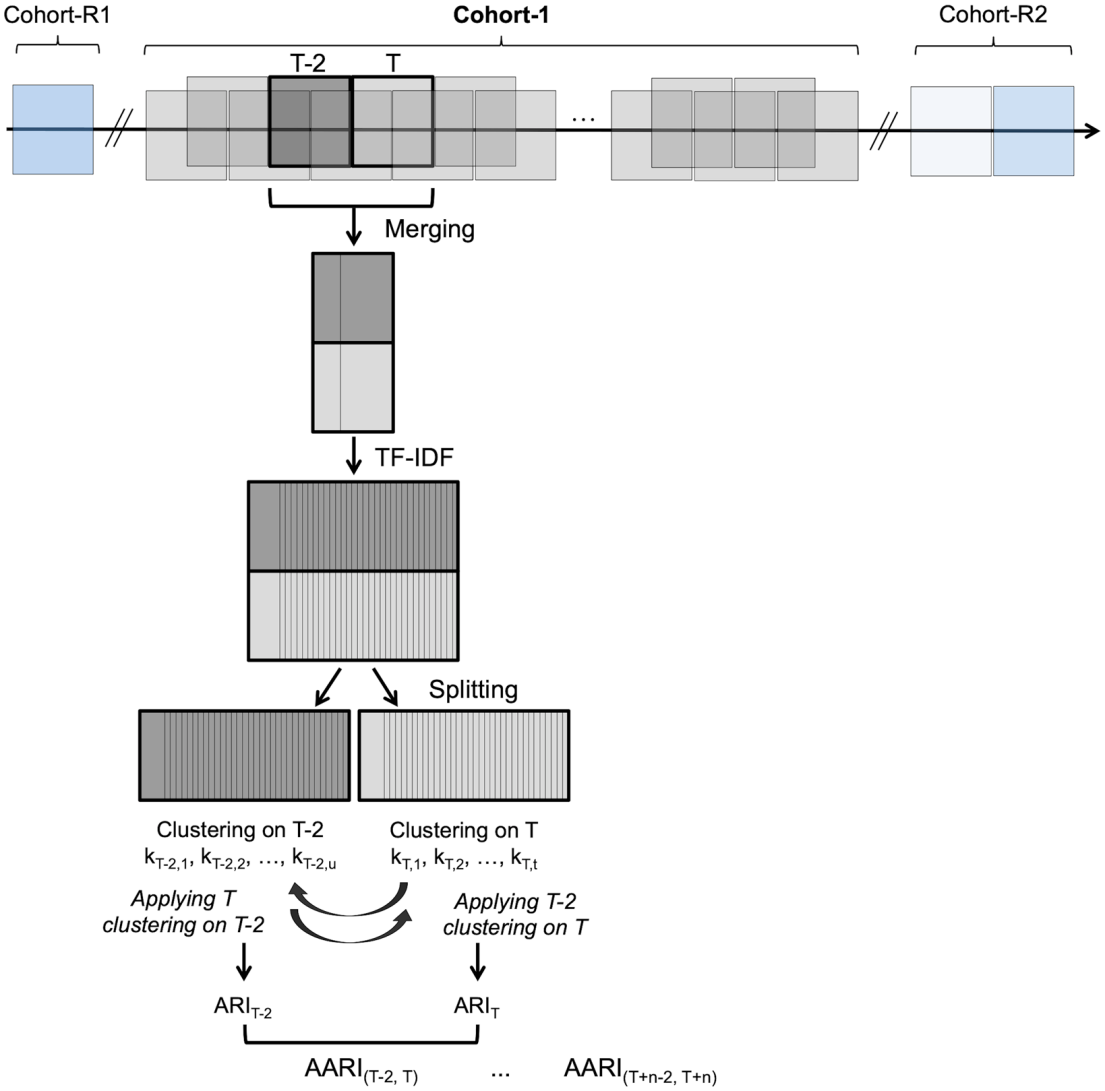
Principle of the text clustering. The full study period from Cohort-1 was split into several time periods of 2 weeks long with an increment of 1 week. Abbreviations: T, a given time period; T-2, a time period corresponding to 2 weeks before T with no overlap; TF-IDF, term frequency–inverse document frequency; ARI, adjusted Rand index; AARI, average adjusted Rand index

The principle of the analysis was as follows (Fig. 2):

- For each time period T of 2 weeks (with an increment of one week), we filtered the N_T_ observations from T and the N_T-2_ observations in reference period T-2.
- We performed a term frequency–inverse document frequency (TF-IDF) analysis on all stemmed nonstop words identified during T and T-2 (n = n_words(T + T-2)_), which enabled the conversion of text to n_words(T + T-2)_ numeric variables (methodology in Supplemental Data S2) [19].
- We extracted the N_T_ observations from T and performed an unsupervised classification based on TF-IDF values, the partition around medoid (PAM) algorithm and the Pearson distance (methodology in Supplemental Data S3) using the “amap,” “cluster,” and “fpc” packages [20]. Hence, we obtained a new cluster variable named K_T_ with t levels (k_T,1_, k_T,2_, …, k_T,t_).
- Similarly, we extracted the observations from T-2 and performed an unsupervised classification. Hence, we obtained another new cluster variable named K_T-2_ with u levels (k_T-2,1_, k_T-2,2_, …, k_T-2,u_).
- We then determined to which cluster K_T-2_ from T-2 each observation from T belonged. Hence, we calculated the Pearson distance between the observation of interest from T and each of the u medoids of the clusters from T-2. Next, we determined the cluster with the smallest distance. Hence, we obtained a new label for the N_T_ observations named K’_T-2’_ with u levels (k_T-2,1_, k_T-2,2_, …, k_T-2,u_).
- Similarly, we determined to which cluster from T each observation from T-2 belonged, and we obtained a new label for the N_T-2_ observations named K’_T’_ with t levels (k_T,1_, k_T,2_, …, k_T,t_).

We repeated this process for each pair of consecutive time periods (T-2, T) from Cohort-1, with 1-week increments.

As a confirmatory analysis, we repeated the same analysis using Cohort-R1 and the last 2 weeks of Cohort-R2 (Cohort-R2’) as references.

### Additional Data Collection

#### Clinical and Radiological Annotations

For all cohorts, we extracted the following information: patient age and sex and CT protocol (i.e., contrast medium injection, body areas covered by CT scans, CT pulmonary angiogram (CTPA)). The nature of the conclusion of the CT reports was prospectively encoded by the emergency radiologists when validating the CT report (categorized as “nonpathological,” “pathological, related to symptoms,” and “pathological, unrelated to symptoms” (i.e., fortuitous)). Of note, “pathological, related to symptoms” did not mandatorily imply COVID-19 lung disease and did not reflect the severity of the pathological findings.

#### Epidemiological Datasets

Epidemiological datasets were retrieved from data.gouv.fr, an open-source platform storing public datasets [10]. We used the daily time series of the Advanced Sanitary Index of flu syndromes (ASI-flu, highly correlated with the incidence of flu syndromes) and the number of positive tests for SARS-CoV-2 across the French territory. We then filtered the observations over the same time periods as Cohort-1, Cohort-R1, and Cohort-R2. It must be noted that the epidemiological datasets and the radiological datasets were not directly matched by patient.

#### Converting to Time Series

For all time periods, we counted the number of stemmed nonstop words related to the main pathological radiological features, namely: (1) consolidation, (2) fibrosis, (3) effusion, (4) nodule, (5) ground glass opacities, (6) lymphadenopathies, (7) crazy paving, and (8) reticulation) and divided it by the number of observations from the time period of interest to obtain their frequency and to understand the iterative unsupervised clusters obtained over time. The raw images corresponding to the CT reports were not reviewed to verify the actual presence of the features. We also counted the percentage of CTPAs, the percentage of pathological examinations, the number of newly confirmed SARS-CoV-2 infections, and the average ASI-flu value.

### Statistical Analyses

Statistical analyses were also performed with R (v4.1.0). All tests were two-tailed. A *P* value < 0.05 was deemed significant. Associations between categorical variables were tested with chi-square tests.

#### Comparing the Similarities of Clusters

For each pair of time periods (T-2, T), the similarity between the K_T_ and K’_T-2’_ clusters (in T) and between the K_T-2_ and K’_T_ (in T-2) clusters were calculated using the adjusted Rand index (ARI) (methodology in Supplemental Data S4) [21], and confidence intervals (CIs) were evaluated using bootstrapping on 1000 replicates using the “pdfCluster” and “boot” packages. Next, we calculated the average ARI value for each pair of time periods (T-2, T) as follows:

- AARI(T-2, T) = 0.5 × [ARI(K_T_ and K’_T-2_ in T) + ARI(K_T-2_ and K’_T_ in T-2)].

Furthermore, we calculated the AARI values between the first and last 2 weeks of Cohort-R2 (i.e., AARI (Cohort-R2, Cohort-R2’)), using Cohort-R1 as a reference (i.e., AARI (Cohort-R1, T) for each T from Cohort-1) and the last 2 weeks of Cohort-R2 as a reference (i.e., AARI (T, Cohort-R2’) for each T from Cohort-1).

#### Explaining Clustering Dissimilarity

Correlations between time series were investigated with the cross correlation function (CCF) (methodology in Supplemental Data S5). Moreover, time series linear regressions between the number of SARS-CoV-2-positive tests, ASI-flu syndromes, and AARI values were performed for different lags. In this comprehensive analysis of AARI values, the explanatory variables were the number of SARS-CoV-2-positive tests and ASI (both provided in epidemiological datasets). The goodness-of-fits were evaluated with the adjusted *R*-squared values (adj-*R*^2^, or coefficient of determination—methodology in Supplemental Data S6) [22].

## Results

### Study Populations (Table 1)

**Table 1:** Characteristics of the main cohort (Cohort-1) and the two reference cohorts (Cohort-R1 and Cohort-R2)

**Characteristics**	**Examinations from Cohort-1**	**Examinations from Cohort-R1**	**Examinations from Cohort-R2**
**Sex**			
Women	6029/13235 (45.6%)	340/845 (40.2%)	1983/4292 (46.2%)
Men	7206/13235 (54.4%)	505/845 (59.8%)	2309/4292 (53.8%)
**Age (years, median [IQR] (range))**	65 [45–78] (0–100)	62 [41–77] (0–99)	71 [57–82] (1–101)
**Pediatrics examination**			
No	12885/13235 (97.4%)	819/845 (96.9%)	4260/4292 (99.3%)
Yes	349/13235 (2.6%)	26/845 (3.1%)	33/4292 (0.8%)
*Unknown age*	1/13235 (0%)	0/845 (0%)	0/4292 (0%)
**CT protocol on chest**			
No injection	2761/13235 (20.9%)	120/845 (14.2%)	1253/4292 (29.2%)
CTPA	5598/13235 (42.3%)	343/845 (40.6%)	2919/4292 (68%)
Injection with arterial phase	706/13235 (5.3%)	53/845 (6.3%)	59/4292 (1.4%)
Injection with portal time	2070/13235 (15.6%)	132/845 (15.6%)	61/4292 (1.4%)
Body scanner	2077/13235 (15.7%)	197/845 (23.3%)	0/4292 (0%)
Multiorgan removal protocol	23/13235 (0.2%)	0/845 (0%)	0/4292 (0%)
**Chest coverage**			
Chest CT only	7699/13235 (58.2%)	416/845 (49.2%)	4292/4292 (100%)
Chest CT + other body area	5536/13235 (41.8%)	429/845 (50.8%)	-
**Pathologic examination**			
No	4180/13235 (31.6%)	275/845 (32.5%)	938/4292 (21.9%)
Yes, related to symptoms	7417/13235 (56%)	475/845 (56.2%)	3014/4292 (70.2%)
Yes, fortuitous—unrelated to symptoms	1638/13235 (12.4%)	95/845 (11.2%)	340/4292 (7.9%)
**No. of involved radiologists**	171	83	173
**No. of examinations per radiologist**			
Mean ± SD	77.4 ± 61	10.2 ± 7.1	24.8 ± 20.6
Median [IQR] (range)	61 [30–108] (2–338)	9 [5–13] (1–40)	19 [11–33] (1–136)

Data are number of patients with percentage in parentheses except for age, the number (no.) of radiologists and the no. of examinations per radiologist

*CTPA* CT pulmonary angiogram, *IQR* interquartile range, *SD* standard deviation

There were 13,235 patients included in Cohort-1 (6,029 women/13,235 [45.6%] patients, median age: 65 years, Q1–Q3 = 45–78), 845 in Cohort-R1 (340 women/845 [40.2%] patients, median age: 62 years, Q1–Q3 = 41–77), and 4292 in Cohort-R2 (1983 women/4292 [46.2%] patients, median age: 71 years, Q1-Q3 = 57–82) (Fig. 1).

Overall, 7417/13,235 (56%) examinations were labeled “pathological, related to symptoms” in Cohort-1 versus 475/845 (56.2%) in Cohort-R1 and 3014/4292 (70.2%) in Cohort-R2 (*P* < 0.0001).

Regarding protocols, 5598/13,235 (42.3%) examinations from Cohort-1 included CTPAs, compared to 343/845 (40.6%) and 2919/4292 (68%) examinations in Cohort-R1 and Cohort-R2 (*P* < 0.0001).

The list of CT devices used across all the partner centers is given in Supplementary Data S7.

### Cluster Comparison Results Over Time

**Fig. 3:**
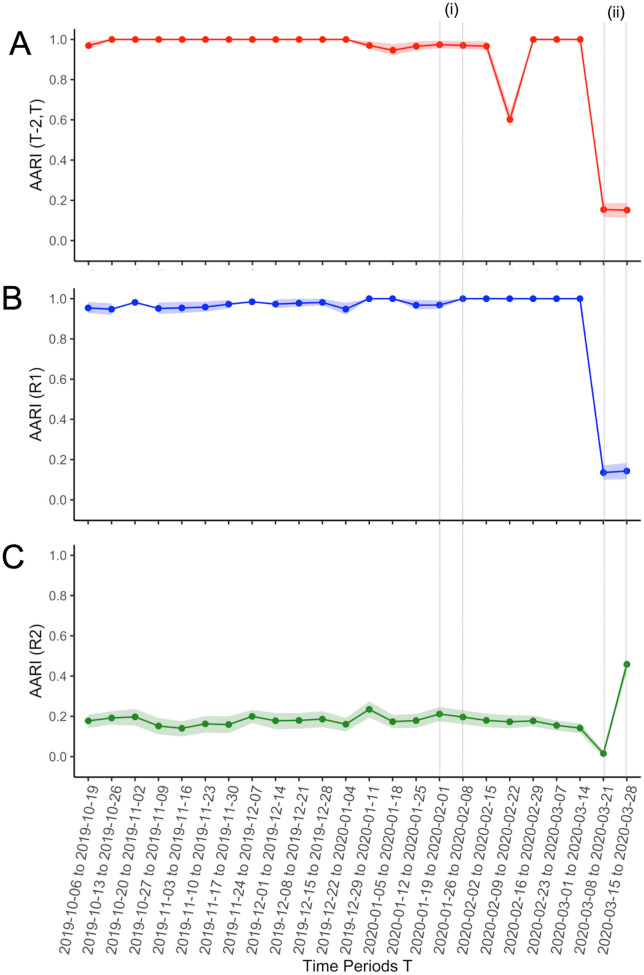
Average adjusted Rand index (AARI) as a function of time in **A** the main, iterative (T, T-2) approach (i.e., evaluating similarities between reports from a given time period T with the reports from 2 weeks before; **B** the (T, R1) approach (i.e., evaluating similarities between reports from a given time period T with the reports from a reference period R1 far before any wave of infection); and **C** the (T, R2) approach (i.e., evaluating similarities between reports from a given time period T with the reports from a reference period R2 during the peak of the 2nd French COVID-19 wave). The vertical dotted lines correspond to (i) the first official cases of SARS-CoV-2 infection in France and (ii) the 1st French lockdown

Regarding the (T-2, T) iterative approach, a strong dissimilarity in text clusters was found for the last two periods from Cohort-1, namely, 2020/03/08 to 2020/03/21 and 2020/03/15 to 2020/03/28 (AARI = 0.154 [95% CI = 0.117–0.186] and AARI = 0.151 [95% CI = 0.114–0.187], respectively) (Fig. 3A). The other AARI values ranged from 0.601 to 1.

Using Cohort-R1 as a reference, the AARI values ranged between 0.948 and 1 for the biweekly periods starting from 2019/10/06 to 2020/03/01. The lowest AARI values were found for the 2020/03/08 to 2020/03/21 period (AARI = 0.135, 95% CI = 0.099–0.171) and the 2020/03/15-to-2020/03/28 period (AARI = 0.143, 95% CI = 0.102–0.183) (Fig. 3B).

Using Cohort-R2 as a reference, the AARI values ranged between 0.015 and 0.234 for the periods from 2019/09/01 to 2020/03/08. The highest similarity was found for the 2020/03/15 to 2020/03/28 period (AARI = 0.458, 95% CI = 0.422–0.492) (Fig. 3C).

Last, the AARI value for the two biweekly time periods from Cohort-R2 was 0.560 (95% CI = 0.534–0.585).

### Analyzing Words from Dissimilar Periods

We investigated which words were increasingly mentioned by analyzing the strongest variations (top 10) in the quantile of the number of quotations during the most dissimilar periods, i.e., from 2020/03/08 to 2020/03/21 and from 2020/03/15 to 2020/03/28 (see Table in Supplemental Data S7).

Notably, the words “crazy” and “paving” were quoted once in Cohort-R1 (from 2019/09/01 to 2019/09/14) versus 79 times from 2020/03/08 to 2020/03/21, and 291 times from 2020/03/15 to 2020/03/28, respectively. In the iterative approach, “crazy” and “paving” were quoted 4 and 10 times the 2 weeks before these two periods of interest (i.e., from 2020/02/23 to 2020/03/07, and from 2020/03/01 to 2020/03/14, respectively).

The root “fibro” (found in “fibrosis” or “fibrotic”) was found in 12 reports in Cohort-R1 compared to 18 reports in the period from 2020/02/23 to 2020/03/07, 322 reports from 2020/03/08 to 2020/03/21, and 1221 reports from 2020/03/15 to 2020/03/28.

### Correlations with Other Time Series

#### Against Epidemiological Data

**Fig. 4:**
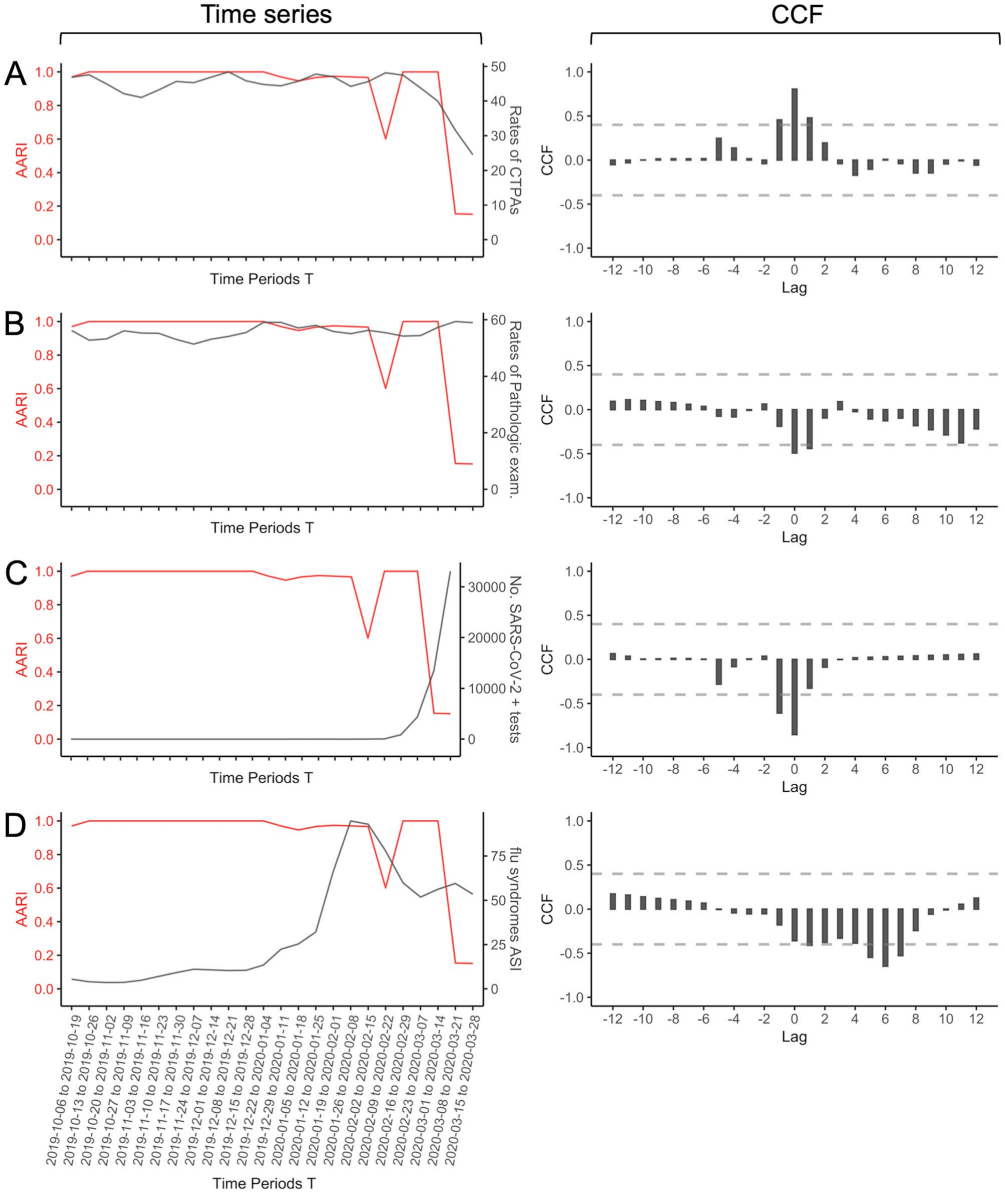
Cross-correlation between AARI(T-2,T) and clinical and epidemiological biweekly time series for **A** the number of C pulmonary angiograms (CTPAs), **B** the number of pathologic examinations related to symptoms, **C** the number of SARS-CoV-2 positive RT-PCRs, and **D** the advanced sanitary index (ASI) for flu syndromes. The plots on the left systematically represent the time series along time, and the plot on the right the cross-correlation function (CCF) results with different lags. Horizontal dashed lines indicate significativity

The biweekly time series related to the two SPF datasets, the rates of CTPAs, and the rates of pathological examinations are shown in Fig. 4, with their CCFs against AARI values based on the iterative approach. Table 2 shows the time lag with significant cross-correlations. The highest significant CCFs were found at lag = 0 for the rates of CTPAs (CCF = + 0.805, *P* = 0.0003), the rates of pathological examinations (CCF = − 0.493, *P* = 0.0211), and the number of positive SARS-CoV-2 tests (CCF = -0.854, *P* = 0.0001) and at lag = + 6 for the ASI-flu value (CCF = − 0.648, *P* = 0.0042, i.e., significant correlations with the AARI values six weeks later).

**Table 2:** Significant lags and correlations obtained between the average adjusted Rand index (AARI) and clinical and epidemiological biweekly time series

**Cross-correlations with**	**Lag**^**a**^	**CCF coefficient**	**Unadjusted *P* value**	**Adjusted *P* value**
Rates of CTPA	− 1	0.456	0.0255	0.0298*
	**0**	**0.805**	< 0.0001	0.0003***
	1	0.478	0.0191	0.0243*
Rates of pathological examinations related to symptoms	**0**	− **0.493**	0.0158	0.0211*
	1	− 0.441	0.0307	0.0331*
Average ASI for flu syndromes	1	− 0.411	0.0440	0.0454*
	5	− 0.547	0.0073	0.0121*
	**6**	− **0.648**	0.0015	0.0042**
	7	− 0.528	0.0097	0.0143*
No. of SARS-CoV-2 positive tests in France	− 1	− 0.609	0.0029	0.0057*
	**0**	− **0.854**	< 0.0001	0.0001***

*P* value are adjusted using the Benjamini–Hochberg procedure. Values in bold correspond to the highest absolute value of the CCF coefficient and its corresponding lag, for each clinical and epidemiological feature of interest

*ASI* advanced sanitary index, *CCF* cross-correlation function, *CTPA* CT pulmonary angiogram, *GGO* ground glass opacities, *no.* number

^*^P < 0.05; **P < 0.005; ***P < 0.001

^a^Lag: correspond to the decay between the two time series. If significance is found at Lag = k between AARI and one of the time series, then, the results should be interpreted as the correlation between the AARI at t—k and the time series at t

#### Against Pathological Radiological Features

**Fig. 5:**
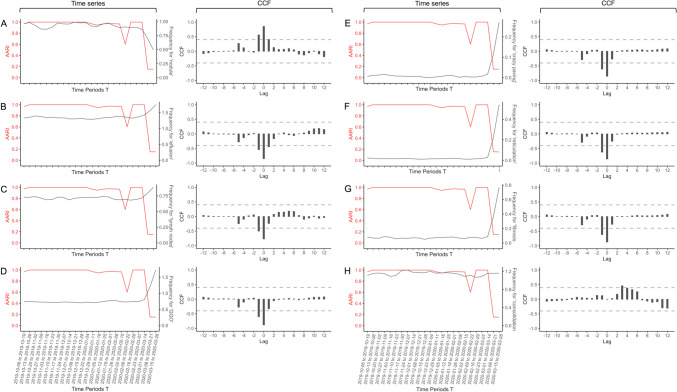
Cross-correlation between AARI(T-2,T) and radiological biweekly time series for the frequency of the words: **A** “nodule,” **B** “effusion,” **C** “lymph node,” **D** “ground glass opacity,” **E** “crazy paving,” **F** “reticulation,” **G** “fibrosis,” and **H** “consolidation.” The plots on the left systematically represent the time series along time, and the plot on the right the cross-correlation function (CCF) results with different lags. Horizontal dashed lines indicate significativity

The eight biweekly time series for the words related to the main radiological features shown on chest CT are shown in Fig. 5, with their CCFs and simple correlation plots against AARI values based on the iterative approach. Table 3 shows the time lag with significant cross-correlations. The highest significant CCFs were found at lag = 0 for nodules (CCF = + 0.851, *P* = 0.0001), effusion (CCF = − 0.848, *P* = 0.0001), lymphadenopathies (CCF = − 0.769, *P* = 0.0005), GGOs (CCF = − 0.882, *P* = 0.0001), crazy paving (CCF = − 0.856, *P* = 0.0001), reticulations (CCF = − 0.856, *P* = 0.0001), and fibrosis (CCF = − 0.871, *P* = 0.0001) and at lag = + 3 for consolidation (CCF = 0.462, *P* = 0.0289).

**Table 3:** Significant lags and correlations obtained between the average adjusted Rand index (AARI) and textual biweekly time series related to the main radiological features described on chest CT

**Cross-correlations with**	**Lag**^**a**^	**CCF coefficient**	**Unadjusted *P* value**	**Adjusted *P* value**
Nodules	− 1	0.558	0.0062	0.0109*
	**0**	**0.851**	< 0.0001	0.0001***
	1	0.409	0.0454	0.0454*
Effusion	− 1	− 0.539	0.0082	0.0129*
	**0**	**− 0.848**	< 0.0001	0.0001***
	1	− 0.441	0.0307	0.0331*
Lymph nodes	− 1	− 0.499	0.0145	0.0202*
	**0**	**− 0.769**	0.0002	0.0005***
GGO	− 1	− 0.6	0.0033	0.0062*
	**0**	**− 0.882**	< 0.0001	0.0001***
Crazy paving	− 1	− 0.611	0.0028	0.0057**
	**0**	**− 0.856**	< 0.0001	0.0001***
Reticulation	− 1	− 0.621	0.0024	0.0057**
	**0**	**− 0.856**	< 0.0001	0.0001***
Fibrosis	− 1	− 0.618	0.0025	0.0057**
	**0**	**− 0.871**	< 0.0001	0.0001***
Consolidation	**3**	**0.462**	0.0237	0.0289*

*P* values are adjusted using the Benjamini–Hochberg procedure. Values in bold correspond to the highest absolute value of the CCF coefficient and its corresponding lag, for each clinical and epidemiological feature of interest

*ASI* advanced sanitary index, *CTPA* CT pulmonary angiogram, *GGO* ground glass opacities

^*^P < 0.05; **P < 0.005; ***P < 0.001

^a^Lag: correspond to the decay between the two time series. If significance is found at Lag = k between AARI and one of the time series, then, the results should be interpreted as the correlation between the AARI at t—k and the time series at t

#### Linear Regressions

The strong decrease in AARI values was aligned with the strong increase in the number of positive SARS-CoV-2 tests (Fig. 4C). A closer look at the time series plot for AARI values identified another local minimum for the 2020/02/09 to 2020/02/22 period (AARI = 0.601), which followed the flu syndrome peak (maximal ASI-flu value = 94.7 for the 2020/01/26 to 2020/02/08 period (i.e., for lag = + 1) and ASI value = 92.9 for the 2020/02/02 to 2020/02/15 period (i.e., for lag = + 2)) (Fig. 4D). Hence, we performed three regressions with lag = 0, + 1, and + 2 for ASI-flu values (Table 4). The highest coefficients of determination was obtained for the model with a lag = + 2 for ASI-flu values (adj-*R*^2^ = 0.921 versus adj-*R*^2^ = 0.856 for lag = 0 and adj-*R*^2^ = 0.769 for lag = + 1). For this best model, the ASI-flu value at lag = + 2, the number of positive SARS-CoV-2 tests and their interaction were significantly correlated with the AARI value (coefficient = − 1.86 × 10^−3^, *P* = 0.0026; coefficient = − 4.75 × 10^−4^, *P* < 0.0001, and coefficient = 8.05 × 10^−5^, *P* < 0.0001, respectively).

**Table 4:** Results of regression modeling between biweekly time series

**Model**	**Characteristics**	**Coefficients (β)**	**S.E**	***P* value**	**Adjusted *R***^**2**^
Model 0	Average ASI-flu (lag = 0)	− 9.53 × 10^−4^	6.63 × 10^−4^	0.1661	0.856
	No. of SARS-CoV-2 positive tests	2.65 × 10^−4^	6.71 × 10^−5^	0.0008***	
	Average ASI-flu (lag = 0) * no. of SARS-CoV-2 positive Tests	− 5.41 × 10^−6^	1.24 × 10^−6^	0.0003***	
	Intercept	1.01	-	-	
Model 1	Average ASI-flu (lag = 1)	− 1.365e^−03^	8.769e^−04^	0.1360	0.769
	No. of SARS-CoV-2 positive tests	− 2.950e^−04^	1.426e^−04^	0.0524	
	Average ASI-flu (lag = 1) * no. of SARS-CoV-2 positive Tests	4.535e^−06^	2.414e^−06^	0.0757	
	Intercept	1.02	-	-	
**Model 2**	Average ASI-flu (lag = 2)	− 1.86 × 10^−3^	5.31 × 10^−4^	0.0026**	**0.921**
	No. of SARS-CoV-2 positive tests	− 4.75 × 10^−4^	6.53 × 10^−5^	< 0.0001***	
	Average ASI-flu (lag = 2) * no. of SARS-CoV-2 positive Tests	8.05 × 10^−6^	1.17 × 10^−6^	< 0.0001***	
	Intercept	1.01	-	-	

Significant P-values are in bold

*ASI-flu* advanced sanitary index for flu syndromes, *no.* number, *SE* standard error

^*^P < 0.05; **P < 0.005; ***P < 0.001

## Discussion

Herein, we proposed an innovative method based on text cleaning, TF-IDF vectorization, unsupervised clustering, and time series analysis to investigate whether the content of radiological reports changed in the beginning of an outbreak of a new emerging disease before the availability of standardized reports specific to this disease and the spread of medical knowledge across the radiological community. Based on the example of the beginning of the COVID-19 pandemic, our results showed that this method was feasible and provided a similarity measure, which was negatively correlated with the incidence of new cases of SARS-CoV-2.

Our method takes advantage of common information and technology tools in teleradiology. As the examinations were performed in several centers scattered across France, these data sampled emergency activity and provided an overview of what was occurring in emergency departments. A prior study highlighted that teleradiological monitoring of the SFR-SIT diagnostic score could approximate the course of the COVID-19 pandemic in France [23]. However, developing such a workflow relying on the SFR-SIT score implies that we already know that a new disease has emerged and its semiology. Herein, our goal was to identify breaks in the content of reports automatically and in an unsupervised manner without a priori information.

The similarity between consecutive clusters shrank in early March 2020 (from an AARI value of 1 to 0.15), which corresponds to the inflexion of positive SARS-CoV-2 tests (i.e., 49 patients across France for the 2020/02/16 to 2020/02/29 period, 4376 for the 2020/02/23 to 2020/03/07 period, 13,510 for the 2020/03/01 to 2020/03/14, and 33,075 for the 2020/03/15 to 2020/03/28 period) [10]. To confirm these results, we replicated the same unsupervised method but with different reference periods. Using Cohort-R1 as a reference, we observed similar variations in AARI values (T, R1), that is, a strong decrease in March 2020. Using Cohort-R2’ as a reference, we observed an increase in the AARI value (T, R2) in March 2020, which means that reports in March 2020 were increasingly similar to reports from the 2nd wave peak, when SFR-SIT-based standardized reports were widely used.

To understand these temporal variations, we investigated associations with simpler textual data (i.e., the frequency of words related to chest CT semiology), the number of CTPAs and pathological examinations, and epidemiological data at different lags. We found strong negative correlations between AARI values (T, T-2) and the number of pathological examinations related to symptoms (CCF coefficient = − 0.493) and the number of positive SARS-CoV-2 tests (CCF coefficient = − 0.853) at lag = 0. Conversely, the number of requests for CTPAs showed a positive correlation (CCF coefficient = + 0.805 at lag = 0) because the relationships among COVID-19 infection, the prothrombotic state and pulmonary embolism were not already known but were described in late April 2020 [24]. To date, only non-contrast-enhanced chest CT scans have been performed for acute respiratory symptoms. Regarding cross-correlations with the main radiological features, the AARI value (T, T-2) was positively correlated with the words “nodules” (CCF coefficient = 0.851) at lag = 0 and “consolidation” (CCF coefficient = 0.462) at lag = 3. Actually, these features were rarely encountered during COVID-19 lung infection and generally due to superinfection [25], whereas nodules and consolidation were routinely found in common bacterial pneumonitis and bronchiolitis seen before the COVID-19 outbreak. Conversely, “GGOs,” “crazy paving,” “reticulations,” and “fibrosis” showed very low CCF coefficients (< − 0.800 for all) at lag = 0, which makes sense considering that these features are typical of COVID-19 infection. However, effusion (either pleural or pericardial) and lymphadenopathies were also negatively cross-correlated at lag = 0, although they are not specific to COVID-19 infection (found in 3 to 17.8% of patients with proven SARS-CoV-2 infection) [25–28]. We explained this by the fact that radiologists could have mentioned these features in their reports but in a negative formula. Finally, linear regression analyses emphasized the strong relationships between AARI and ASI-flu values (taking the value 2 weeks before) and the number of positive SARS-CoV-2 tests (at lag = 0). Indeed, an Adj-*R*^2^ value of 0.921 corresponds to an excellent fit. Regarding ASI-flu values, the best fit obtained with this lag can be explained by the entanglement with the end of the flu epidemic in France and the confusion with flu-like symptoms due to COVID-19 exposure occurring 1–2 weeks before clinical worsening requiring a visit to the emergency department.

Future researches could investigate whether this method could prospectively detect the appearance of new SARS-CoV-2 variants or new infectious diseases that would be responsible for pathological radiological features (for instance: infectious colitis or meningitis). In case of breaks in the content of radiological reports (as measured with AARI) at a given time period, the reports and their corresponding images from this time period could be reviewed in details to explain the dissimilarity, and secondarily correlated to geographical, clinical, and biological data of those patients with the help of public health agencies. Furthermore, we believe that correlating radiological time series (such as the raw numbers of normal and pathological imaging per imaging modality per time unit) with economical data could provide relevant information to better anticipate the economical impact of emerging or resurging diseases on hospitals and to better anticipate human and technical resources [29].

Our study has limitations. First, other NLP methods could have been used. The bag-of-words approach and TF-IDF vectorization are classically used in NLP but do not allow us to account for positive or negative formulas. We used PAM and the Pearson distance, as they are robust and usually effective, but other clustering algorithms (such as k-means and HDBSCAN) and distance metrics are available. It is also possible to perform unsupervised clustering on latent layers of autoencoder neural networks or to use latent Dirichlet allocation, which may be more sensitive to detect new trends earlier and in smaller groups of patients [30, 31]. Second, we performed our proof-of-concept demonstration at the beginning of the COVID-19 pandemic, but this method should be confirmed prospectively. Third, it must be noted that the CT reports were not retrospectively reviewed as we used the chest CT reports consecutively performed by radiologists during their on-call duty, in the real-life setting, and provided to emergency physicians. Consequently, it is possible that radiologists missed some pathological findings on chest CT (such as small area of subpleural GGO), especially at the beginning of the COVID-19 outbreak. Actually, it would be hardly feasible to retrospectively review and annotate thousands of CT images and CT reports and we believe that this is an inherent limitation of macroscopic studies performed at the population level. Fourth, various CT devices were used for the CT acquisitions over the partner centers and the study periods, which could have influenced the image quality and the reports.

## Conclusion

In conclusion, we proposed a method to operate large databases of radiological reports routinely collected in practice. Iteratively and automatically assessing the dissimilarities between radiological reports from consecutive periods could help detect variations in the observations made by radiologists, which could have several applications, such as monitoring emerging diseases or any public health issue.

### Supplementary Information

Below is the link to the electronic supplementary material.

Supplementary file1 (DOCX 60 KB)

## Data Availability

Raw data and the R code used to generate the results and figures are available from the corresponding author under reasonable request.

## Abbreviations

AARI: Average adjusted Rand index
ASI-flu: Advanced sanitary index for flu syndromes
CCF: Cross-correlation function
CI: Confidence interval
COVID-19: Coronavirus disease 2019
CTPA: Computed tomography pulmonary angiogram
GGO: Ground glass opacities
NLP: Natural language processing
PAM: Partition around medoids
RT-PCR: Reverse transcriptase polymerase chain reaction
SARS-CoV-2: Severe acute respiratory syndrome coronavirus 2
SFR-SIT: French Society of Radiology–French Society of Thoracic Imaging
TF-IDF: Term frequency–inverse document frequency

## References

[1] Cai T, Giannopoulos AA, Yu S (2016). Natural Language Processing Technologies in Radiology Research and Clinical Applications. Radiographics..

[2] Chen P-H (2020). Essential Elements of Natural Language Processing: What the Radiologist Should Know. Acad Radiol..

[3] Casey A, Davidson E, Poon M (2021). A systematic review of natural language processing applied to radiology reports. BMC Medical Informatics and Decision Making..

[4] Crombé A, Seux M, Bratan F (2022). What Influences the Way Radiologists Express Themselves in Their Reports? A Quantitative Assessment Using Natural Language Processing. J Digit Imaging..

[5] Hassard F, Bajón-Fernández Y, Castro-Gutierrez V (2023). Wastewater-based epidemiology for surveillance of infectious diseases in healthcare settings. Curr Opin Infect Dis..

[6] Sharkey ME, Kumar N, Mantero AMA (2021). Lessons learned from SARS-CoV-2 measurements in wastewater. Sci Total Environ..

[7] Huang C, Wang Y, Li X (2020). Clinical features of patients infected with 2019 novel coronavirus in Wuhan, China. Lancet..

[8] Chung M, Bernheim A, Mei X (2020). CT Imaging Features of 2019 Novel Coronavirus (2019-nCoV). Radiology..

[9] Song F, Shi N, Shan F (2020). Emerging 2019 Novel Coronavirus (2019-nCoV) Pneumonia. Radiology..

[10] Dashboard COVID-19 from the French government: https://www.gouvernement.fr/info-coronavirus/carte-et-donnee. Accessed Jan 2023

[11] Standardized Report for non-contrast-enhanced chest CT according to the French Society of Radiology: SFR e-Bulletin. 2020; https://ebulletin.radiologie.fr/actualites-covid-19/compte-rendu-tdm-thoracique-iv. Accessed Jan 2023

[12] Lassau N, Ammari S, Chouzenoux E (2021). Integrating deep learning CT-scan model, biological and clinical variables to predict severity of COVID-19 patients. Nat Commun..

[13] Das S, Ayus I, Gupta D: A comprehensive review of COVID-19 detection with machine learning and deep learning techniques. Health Technol (Berl). 2023; 1–14. 10.1007/s12553-023-00757-z PMC10244837 37363343

[14] Wang M, Xia C, Huang L (2020). Deep learning-based triage and analysis of lesion burden for COVID-19: a retrospective study with external validation. Lancet Digit Health..

[15] Li L, Qin L, Xu Z (2020). Using Artificial Intelligence to Detect COVID-19 and Community-acquired Pneumonia Based on Pulmonary CT: Evaluation of the Diagnostic Accuracy. Radiology..

[16] Chung J, Kim D, Choi J (2022). Prediction of oxygen requirement in patients with COVID-19 using a pre-trained chest radiograph xAI model: efficient development of auditable risk prediction models via a fine-tuning approach. Sci Rep..

[17] Li MD, Wood PA, Alkasab TK, Lev MH, Kalpathy-Cramer J, Succi MD (2021). Automated tracking of emergency department abdominal CT findings during the COVID-19 pandemic using natural language processing. The American Journal of Emergency Medicine..

[18] Wickham H, Averick M, Bryan J (2019). Welcome to the Tidyverse. Journal of Open Source Software..

[19] Sparck Jones K (1972). A STATISTICAL INTERPRETATION OF TERM SPECIFICITY AND ITS APPLICATION IN RETRIEVAL. Journal of Documentation..

[20] Partitioning Around Medoids (Program PAM): In: Finding Groups in Data. John Wiley & Sons, Ltd, 1990. p. 68–125.

[21] Hubert L, Arabie P (1985). Comparing partitions. Journal of Classification..

[22] Hyndman RJ, Khandakar Y (2008). Automatic Time Series Forecasting: The forecast Package for R. Journal of Statistical Software..

[23] Crombé A, Lecomte J-C, Banaste N (2021). Emergency teleradiological activity is an epidemiological estimator and predictor of the covid-19 pandemic in mainland France. Insights Imaging..

[24] Leonard-Lorant I, Severac F, Bilbault P (2021). Normal chest CT in 1091 symptomatic patients with confirmed Covid-19: frequency, characteristics and outcome. Eur Radiol..

[25] Nivet H, Crombé A, Schuster P (2021). The accuracy of teleradiologists in diagnosing COVID-19 based on a French multicentric emergency cohort. Eur Radiol..

[26] Wong HYF, Lam HYS, Fong AH-T, et al.: Frequency and Distribution of Chest Radiographic Findings in Patients Positive for COVID-19. Radiology. 2020; 296:E72–E78. 10.1148/radiol.2020201160 PMC7233401 32216717

[27] Wang Y, Dong C, Hu Y (2020). Temporal Changes of CT Findings in 90 Patients with COVID-19 Pneumonia: A Longitudinal Study. Radiology..

[28] Caruso D, Zerunian M, Polici M, et al.: Chest CT Features of COVID-19 in Rome, Italy. Radiology. 2020; 201237. 10.1148/radiol.2020201237 PMC7194020 32243238

[29] Lang M, Yeung T, Mendoza DP (2020). Imaging Volume Trends and Recovery During the COVID-19 Pandemic: A Comparative Analysis Between a Large Urban Academic Hospital and Its Affiliated Imaging Centers. Acad Radiol..

[30] Blei DM, Ng AY, Jordan MI (2003). Latent dirichlet allocation. J Mach Learn Res..

[31] Hahsler M, Piekenbrock M, Doran D: **dbscan** : Fast Density-Based Clustering with *R*. J Stat Soft. 2019; 91:.

