# Supplementary material for: Using the Textual Content of Radiological Reports to Detect Emerging Diseases: A Proof-of-Concept Study of COVID-19
Source: J Imaging Inform Med. 2024 Jan 12;37(2):620–32. doi: 10.1007/s10278-023-00949-z (PMC11031522; doi:10.1007/s10278-023-00949-z)
Supplement: Supplementary file 1 — Supplementary file1 (DOCX 60 KB) [file 10278_2023_949_MOESM1_ESM.docx]

**SUPPLEMENTAL MATERIAL**

**SUPPLEMENTAL DATA S1.** Steps of the text pre-processing applied on the chest analysis paragraph of the results of the included radiological reports.

First, for all cohorts, the text was converted to lower case, and the following transformations were performed:

- French accents, which are present upon some vowels (for instance ‘é’, ‘ê’, ‘è’ or ‘ë’), were removed, and the special character ‘ç’ was changed to ‘c’ as a frequent source of spelling mistakes;

- Punctuations, indentations, dates and numbers were removed;

- Symbols and abbreviations were expanded to their full form (i.e., ‘ > ’ to ‘superior’; ‘ < ’ to ‘inferior’; ‘*’ and ‘x’ to ‘multiply’; ‘/’ to ‘over’; and ‘ = ’ to ‘equal’).

Second, the text was tokenized by parsing the sentences into single units, herein unique words.

Third, we used the French dictionary of stop words provided with the ‘proustr’ package (https://github.com/ColinFay/proustr) to remove stop words (n=643 unique stop words after applying the same text processing as above). Stop words are frequent words responsible for irrelevant noise (for instance, ‘a’, ‘an’, ‘and’, ‘the’...etc.).

Fourth, we applied stemming to the remaining words, which consists in solely keeping the root of each word thanks to the algorithm for French provided by the ‘SnowballC’ package (https://github.com/cran/SnowballC).

**SUPPLEMENTAL DATA S2.** Principle of term frequency – inverse document frequency (TF-IDF) analysis.

The aim of TF-IDF analysis is to quantify the importance of a given word in a document (herein, a radiological report) in a collection of documents (herein, a corpus of radiological reports selected per time period). It increases as the number of times the given word appears in the document is high, and is offset by the number of documents in the corpus containing this word.

In this study, TF-IDF is calculated for each stemmed non-stop word « w » and for each radiological report « d » from a collection of document « D ». It corresponds to the product of the term frequency (TF) of the word « w » within the document « d » multiplied by the inverse document frequency (IDF), which is the logarithmically scaled inverse fraction of the documents from « D » that contain the words, as follows:

$$TF-IDF\left( w,d,D \right)= TF\left( w,d \right)\times IDF\left( w,D \right)$$

i.e.,

$$TF-IDF\left( w,d,D \right)= \frac{count\left( w,d \right)}{total no. of words in d}\times log(\frac{total no. of documents in D}{no. of documents where w appears})$$

**References:**

- Karen Spärck Jones, « A statistical interpretation of term specificity and its application in retrieval », Journal of Documentation, vol. 28, no 1,‎ 1972, p. 11–21 (DOI 10.1108/eb026526)

**SUPPLEMENTAL DATA S3.** Clustering methodology using partition around medoids (PAM) and the Pearson distance

The k-medoids correspond to a partitioning method which is more robust to outliers than the k-means. The aim of the k-medoids is to find k groups (C1, C2, ..., Ck) in a dataset with the particularity that the central point of each group (or medoid) is a real observation from the dataset. This average dissimilarity of the medoid of a given group Ci with all the other observation from the group Ci should be the smallest possible.

The PAM algorithm proposed by Kaufman and Rousseeuw enables to solve the problem of the k-medoids (*Kaufman and Rousseeuw, 1990*). It is implemented in the ‘Cluster’ R package and requires to compute first the dissimilarity between all the observations.

In this study, each observation (i.e., each radiological report) is described by a vector of TF-IDF values for each possible stemmed non-stop word encountered in the corpus.

Herein, we used the Pearson distance to measure the dissimilarity between each pair of observation depending on its coordinates in the TF-IDF space, defined as follows:

Let’s note:

- N_W_ the total number of stemmed non-stop word encountered in the reports extracted during the time period of interest (i.e., T U T+2).

- word-1, word-2, ..., word-N_W_ the N_W_ stemmed non-stop words encountered during this time period

- Report-i and Report-j two distinct radiological reports during this time period, which can be described as follows in the TF-IDF space

Report-i = {TF-IDF(word-1,i), TF-IDF(word-2,i), ..., TF-IDF(word-N_W_,i)}

Report-i = {TF-IDF(word-1,j), TF-IDF(word-2,j), ..., TF-IDF(word-N_W_,j)}

Average(TF-IDF, i) the average of the N_W_ TF-IDF values of Report-i

Average(TF-IDF, j) the average of the N_W_ TF-IDF values of Report-j.

Hence, the dissimilary (or distance) between Report-i and Report-j (named Dis(i,j)) according the Pearson distance is calculated as follows (*Kaufman and Rousseeuw, 1990*):

$$Sii= \sum_{n=1}^{N_{W}} {(TF-IDF\left( word-n,i \right) -Average(TF-IDF,i))}^{2}$$

$$Sjj= \sum_{n=1}^{N_{W}} {(TF-IDF\left( word-n,j \right) -Average(TF-IDF,j))}^{2}$$

$$Si,j= \sum_{n=1}^{N_{W}} \left[ TF-IDF\left( word-n,i \right) -Average\left( TF-IDF,i \right) \right]\times\left[ TF-IDF\left( word-n,j \right) -Average\left( TF-IDF,j \right) \right]$$

$$Dis\left( i,j \right)=\frac{1}{2} \times[1- \frac{Si,j}{\sqrt{Sii \times Sjj}}]$$

We apply this formula to all possible pair of reports in the corpus to obtain the dissimilarity matrix.

We then applied the PAM algorithm for various possible numbers k of clusters (from 1 to 10) and we selected the optimal k as the one maximizing the silhouette coefficients on the silhouette plot (Rousseeuw, 1987).

**References:**

- Leonard Kaufman et Peter J. Rousseeuw, « Partitioning Around Medoids (Program PAM) », Wiley Series in Probability and Statistics,‎ 8 mars 1990 (ISBN 978-0-470-31680-1, DOI 10.1002/9780470316801.ch2)

- Peter J. Rousseeuw, « Silhouettes: A graphical aid to the interpretation and validation of cluster analysis », Journal of Computational and Applied Mathematics, vol. 20,‎ 1er novembre 1987, p. 53–65 (ISSN 0377-0427, DOI 10.1016/0377-0427(87)90125-7

**SUPPLEMENTAL DATA S4.** Principle of adjusted Rand index (ARI)

The aim of ARI is to measures the similarity between two classifications (or two partitions) of a same set of observations. In our setting, for a given time period (T-2 or T) we aimed at calculating the similarity between two grouping, namely: K_T_ and K’_T-2_ during the period T and _KT-2_ and K’_T_ in T-2.

In general, let’s note:

- N the total number of observation in the dataset

- P and P’ the two partitions we want to evaluated the similarity

- Ni the number of observations in the group Ci from P

- Nj the number of observations in the group C’j from P’

-Ni,j the number of observations of the group C’j from P’ assigned to the group Cj in P

Hence, ARI for P and P’ is given by the following formula:

$$ARI\left( P,P^{'} \right)= \frac{\sum_{i,j} \left( \begin{matrix} N_{i,j} \\ 2 \end{matrix} \right)-[\sum_{i} (\begin{matrix} N_{i} \\ 2 \end{matrix})\times\sum_{j} (\begin{matrix} N_{j} \\ 2 \end{matrix})]/(\begin{matrix} N \\ 2 \end{matrix})}{\frac{1}{2}\times\left[ \sum_{i} \left( \begin{matrix} N_{i} \\ 2 \end{matrix} \right)+\sum_{j} \left( \begin{matrix} N_{j} \\ 2 \end{matrix} \right) \right]- [\sum_{i} (\begin{matrix} N_{i} \\ 2 \end{matrix})\times\sum_{j} (\begin{matrix} N_{j} \\ 2 \end{matrix})]/(\begin{matrix} N \\ 2 \end{matrix})}$$

A value close to 0 indicates that the similarity is the same as random. The maximal ARI value is 1 for exactly the same partitions.

**References:**

L. Hubert and P. Arabie (1985) Comparing partitions, Journal of Classification, 2, 193-218.

**SUPPLEMENTAL DATA S5.** Principle of cross correlation function (CCF)

The aim of the CCF is to identify linear relationships between lagged values of two time series (Derrick et al., 2004).

Let’s name:

- x(t) and y(t) two time series with n observations (i.e., n time points), and SD_x(t)_ and SD_y(t)_ their standard deviation

- k: the lag (or decay) between two times

Then, the following cross-correlation coefficient is calculated for each k lag:

$$r\left( x+k,y \right)=[\frac{1}{n}\times\sum_{t=1}^{n-k} (y\left( t \right)-\bar{y})\times(x\left( t \right)-\bar{x})]\times\frac{1}{\sqrt{{SD}_{x(t)}\times{SD}_{y(t)}}}$$

r is significant if above or below $-\frac{1}{n}\pm2/\sqrt{n}$.

**References:**

- Derrick TR, Thomas JM (2004) Time series analysis: the cross‐correlation function. Human Kinetics Publishers, Champaign, pp 189–205

- Aragon Y, ‘Series temporelles avec R » EDP sciences edition pratique R, 2016

**SUPPLEMENTAL DATA S6.** Linear regression, AARI(t) and R^2^ metrics.

In order to understand the relationships between the following time series: AARI, no. of positive SARS-CoV-2 tests, ASI for flu and their interaction, time series linear regressions were achieved with different k lags identified on time series plots and cross-correlation analysis.

Regarding time series, a regression model can be written as follows:

$$y\left( t \right)= \beta_{0}+\beta_{1} \times x_{1}\left( t \right)+\beta_{2} \times x_{2}\left( t \right)+\ldots+\beta_{n} \times x_{n}\left( t \right)+\varepsilon(t)$$

Where y(t) is the time series to predict (i.e., the outcome), xi(t) are the predictors with βi their coefficient (1 ≤ i ≤ n), and ε(t) is a random error or deviation from the straight line.

In our example, the equation could be simplified as follows:

$$AARI\left( t \right)=\beta_{0}+\beta_{1} \times ASI\left( t\pm k \right)+\beta_{2} \times No. SARS CoV 2\left( t\pm k' \right)+\beta_{3} \times ASI\left( t\pm k \right) \times No. SARS CoV 2\left( t\pm k' \right)+\varepsilon(t)$$

Where k and k’ are potential lags to investigate

We used the ‘TSLM’ function from the ‘forecast’ R package.

We then calculated the adjusted R-squared (or determination coefficient) to evaluate the goodness of fit, which measures the percentage of variance explains by the model over the total variance of the data, as follows:

Let’s name:

- $\hat{y}$ the predictions from the linear regression model

- y the real value of the predictor

- N the total number of observations

- K the total number of explanatory variables (including intercept and interactions)

$${adjusted-R}^{2}=1-(1- \frac{\sum_{i=1}^{N} \left( \hat{y}_{i}-\bar{y} \right)^{2}}{\sum_{i=1}^{N} \left( y_{i}-\bar{y} \right)^{2}})\times\frac{N-1}{N-K}$$

Adjusted R-squared ranges between 0 (worst model) to 1 (perfect model)

Reference

- Karch, Julian (2020-09-29). "Improving on Adjusted R-Squared". Collabra: Psychology. 6 (45). doi:10.1525/collabra.343

**SUPPLEMENTAL DATA S7.** Types of CT scanners involved in the study

| **CT scanner name and manufacturer** |
| --- |
| Canon Aquilion Prime SP, Canon Medical |
| Canon Aquilion ONE, Canon Medical |
| Canon Aquilion Lightning, Canon Medical |
| GE Optima CT540, GE Healthcare |
| GE Optima CT660, GE Healthcare |
| GE Revolution EVO, GE Healthcare |
| GE Revolution Maxima, GE Healthcare |
| GE Revolution Ascend, GE Healthcare |
| GE Revolution Frontier, GE Healthcare |
| GE Discovery RT, GE Healthcare |
| GE Brightspeed 16B, GE Healthcare |
| GE BrightSpeed Elite CT, GE Healthcare |
| Philips Incisive CT, Philips healthcare |
| Philips Ingenuity 64, Philips healthcare |
| Philips Ingenuity 128, Philips healthcare |
| Philips Ingenuity Core, Philips healthcare |
| Siemens Somatom Definition AS 64, Siemens Healthineers |
| Siemens Somatom Definition AS 128, Siemens Healthineers |
| Siemens Somatom Definition Edge 128, Siemens Healthineers |
| Siemens Somatom Edge Plus, Siemens Healthineers |
| Siemens Somatom Flash, Siemens Healthineers |
| Siemens Fluorospot Compact FD, Siemens Healthineers |
| Siemens Somatom Go.Top, Siemens Healthineers |
| Siemens Somatom Perspective, Siemens Healthineers |

**SUPPLEMENTAL DATA S8.** Words gaining importance in the two time periods with the highest dissimilarity compared to their reference (two weeks before)

| **Week T** | **Words/stem** | **During the T time period** | |  | **During the T-2 time period** | |  | **Change in quantile between T-2 and T^§^** |  | **During the R1 time period** | **During the R2* time period** |
| --- | --- | --- | --- | --- | --- | --- | --- | --- | --- | --- | --- |
|  |  | **NQ** | **Quantile** |  | **NQ** | **Quantile** |  |  |  | **NQ** | **NQ** |
| from 2020-03-08 to 2020-03-21 | covid | 70 | 0.951 |  | 2 | 0.604 |  | 0.347 |  | 0 | 45 |
|  | viral | 28 | 0.907 |  | 2 | 0.604 |  | 0.303 |  | 1 | 31 |
|  | paving | 79 | 0.953 |  | 4 | 0.699 |  | 0.255 |  | 1 | 36 |
|  | crazy | 79 | 0.953 |  | 4 | 0.699 |  | 0.255 |  | 1 | 36 |
|  | infect | 66 | 0.950 |  | 4 | 0.699 |  | 0.251 |  | 0 | 90 |
|  | traction | 39 | 0.929 |  | 4 | 0.699 |  | 0.230 |  | 0 | 189 |
|  | endobronch | 36 | 0.924 |  | 7 | 0.774 |  | 0.150 |  | 3 | 22 |
|  | reticul | 288 | 0.978 |  | 16 | 0.868 |  | 0.110 |  | 19 | 659 |
|  | fibro | 322 | 0.980 |  | 18 | 0.879 |  | 0.102 |  | 12 | 833 |
|  | debut ('*start*') | 318 | 0.980 |  | 18 | 0.879 |  | 0.101 |  | 16 | 76 |
| from 2020-03-15 to 2020-03-28 | mineur ('*minor*') | 137 | 0.963 |  | 0 | 0.285 |  | 0.678 |  | 1 | 314 |
|  | unilateral | 51 | 0.931 |  | 1 | 0.540 |  | 0.391 |  | 0 | 51 |
|  | bacterien ('*bacterial*') | 30 | 0.902 |  | 1 | 0.540 |  | 0.362 |  | 0 | 35 |
|  | traction | 125 | 0.960 |  | 5 | 0.754 |  | 0.206 |  | 0 | 189 |
|  | majeur ('*major*') | 71 | 0.946 |  | 5 | 0.754 |  | 0.192 |  | 9 | 110 |
|  | paving | 291 | 0.977 |  | 10 | 0.837 |  | 0.140 |  | 1 | 36 |
|  | crazy | 291 | 0.977 |  | 10 | 0.837 |  | 0.140 |  | 1 | 36 |
|  | covid | 138 | 0.964 |  | 9 | 0.826 |  | 0.137 |  | 0 | 45 |
|  | topograph ('*location*') | 58 | 0.938 |  | 8 | 0.813 |  | 0.125 |  | 7 | 40 |
|  | infect | 117 | 0.959 |  | 11 | 0.844 |  | 0.114 |  | 0 | 90 |

NOTE.- Abbreviations: NQ: number of quotations, quantile: quantile of the value of NQ with regards to all the other stemmed non-stop words after pooling the (T-2 and T) time periods.

§: The change in quantile between T-2 and T simply corresponds to the absolute difference of quantile.
